# Psychological distress and cancer pain: Results from a controlled cross-sectional survey in China

**DOI:** 10.1038/srep39397

**Published:** 2017-01-11

**Authors:** Xiao-Mei Li, Wen-Hua Xiao, Ping Yang, Hui-Xia Zhao

**Affiliations:** 1 Department of Medical Oncology, Chinese PLA General Hospital, Beijing, No. 28, Fuxing Road, Haidian district, Beijing 100853, China; 2 Department of Medical Oncology, the First Affiliated Hospital of Chinese PLA General Hospital, Beijing 100048, China; 3 Department of Medical Oncology, PLA Navy General Hospital, Beijing 100048, China

## Abstract

We evaluated the pain associated with cancer and its impact on pain management, anxiety, and depression in Chinese patients using a controlled cross-sectional study. One hundred and twenty-six cancer outpatients were evaluated from January 2012 to June 2014; 64 reported pain and 62 did not. Patients with cancer eligible for this study were older than 18 years and able to effectively communicate with medical personnel. Patients were administered a questionnaire regarding their medical status. The information collected was used along with patient charts to complete a socio-demographic and clinical characteristic summary for each patient. Results showed that patients who reported pain had mean State-Trait Anxiety Inventory (STAI) scores of 46.38 for state anxiety and 44.64 for trait anxiety, as well as a mean BDI (Beck Depression Inventory) score of 19.17. The pain-free patient group had mean STAI scores of 40.73 for state anxiety and 42.87 for trait anxiety, and a mean BDI score of 15.35. In conclusion, patients who reported pain were more prone to anxiety and depression, with pain severity being a strong predictor of anxiety. Adequate pain assessment and adjustment proved necessary for pain management.

Pain is one of the most common symptoms for patients with cancer. Despite established cancer pain management guidelines, more than 50% of patients do not obtain adequate pain relief, and undertreatment of pain is still a widespread problem worldwide^1^,^2^,^3^.

In the early 1990 s, Wang, *et al*.^4^ conducted a study in Beijing in which they demonstrated the reliability and validity of the Chinese version of the Brief Pain Inventory (BPI-C). Cancer pain and pain treatment were also assessed using the newly developed BPI-C and Pain Management Index (PMI). In their study, 67% of cancer pain was undertreated and Chinese patients reported higher levels of pain severity and pain interference compared with patients in similar studies conducted at the same time (1991–1992) in the United States and France.

Two decades have passed since then and we believe that overall pain control has been greatly improved in China, given the gradually increased availability of opioids and better attitudes and skills of physicians regarding cancer pain treatment. However, no published study has reassessed cancer pain and pain treatment in Mainland China in recent years; thus, specific strategies for further improvement remain ambiguous. We have evaluated and analyzed the pain experienced by Chinese patients with cancer and its management. Furthermore, we assessed the level of anxiety and depression in patients who reported experiencing pain.

Anxiety and depression are the most common psychological symptoms in patients with cancer pain^5^,^6^,^7^. Inadequate pain control contributes to increased prevalence and severity of these symptoms and may increase the complexity and difficulty of pain management. Satisfactory cancer pain relief requires clear recognition of the negative impacts of pain on patient’s psychological functions. However, very few studies have been conducted that evaluate anxiety and depression in patients with cancer pain in Mainland China. Little is known about how and to what extent cancer pain affects patients’ anxiety and depression. Therefore, the second objective of this study was to evaluate the levels of anxiety and depression in patients with pain.

Since previous studies have demonstrated that patients with cancer without pain also presented with higher levels of anxiety and depression compared with healthy controls^8^,^9^,^10^, we recruited pain-free patients with cancer at the same time as a control group. Furthermore, univariate and multivariate analyses were conducted to determine the extent to which pain-related factors, as well as socio-demographic and clinical characteristics, were correlated with anxiety and depression.

## Patients and Methods

### Study Design

A controlled cross-sectional study was conducted at the Medical Oncology Department of the First Affiliated Hospital of Chinese PLA General Hospital, a 1000-bed tertiary teaching hospital in Beijing, China. The study protocol was approved by the Ethics Committee and the Institutional Review Board of the First Affiliated Hospital of Chinese PLA General Hospital. Informed consent was obtained from each patient participating in this study after explaining the risks and benefits of the study in an ethical manner. The methods were carried out in accordance with the approved guidelines. All experiments were performed in accordance with the Declaration of Helsinki.

Each group had an equal number of outpatients, recruited based on the presence or absence of cancer pain. The authors of this study trained physicians regarding patient enrollment based on complaints, symptoms, and imaging data. Patients with pain were enrolled in the pain group and patients without pain were enrolled in the non-pain group at a 1:1 ratio. Pain status was assessed verbally by asking if the patient experienced pain during the previous week and patients were categorized into the respective groups. Pain assessments were performed with the pain-group patients, evaluating intensity, pattern, and relief.

Patients who met the following criteria were considered eligible for inclusion: (1) had received a cancer diagnosis, with no limitation for types of cancer or staging, and no limitation for cancer therapy status; (2) were over 18 years of age; (3) were conscious and able to read, write, and communicate. Patients who had a lifetime history of psychopathology or cognitive impairment were excluded.

### Study Procedures

The study was conducted from January 2012 to June 2014. In total, 130 patients with cancer were enrolled in the study: 65 patients with, and 65 patients without pain. Of these patients, 126 were deemed eligible to participate in the study and were evaluated (64 with pain and 62 without pain). One patient in the pain group and 3 in the pain-free group were excluded due to their apparently contradictory or inconsistent responses on the questionnaires. Patients were enrolled into either the pain or pain-free groups according to their pain status.

After informed consent had been obtained, an investigator administrated the questionnaires to both groups of patients. Patients in the pain group received the pain assessment, the BPI-C. Patients in the pain-free group did not receive the BPI-C because their pain intensity and duration would result in a score of 0. The research staff extracted data from the patient’s medical records and completed a form measuring socio-demographic and clinical characteristics, such as staging and tumor response. We observed a significantly decreased Eastern Cooperative Oncology Group Performance Status (ECOG PS) in the pain group (*p* = 0.03), and a significantly higher proportion of patients who were not currently receiving anticancer treatment (*p* = 0.01) (Table 1).

### Statistical Analysis

All statistical analyses were conducted using the Statistical Package for the Social Sciences (SPSS) for Windows 17.0 (SPSS Inc., Chicago, USA). Statistical significance was based on two-sided tests evaluated with 95% confidence intervals. Continuous variables were expressed as means and standard deviations (SD) and were evaluated using the Student’s t-test and one-way analysis of variance (ANOVA). Categorical variables were expressed as numbers and percentages and were evaluated using the Fisher’s exact test and Chi-square test. Mean STAI-S, STAI-T and BDI-II scores of the two groups were analyzed for significant differences. A cutoff value of 14 on the BDI-II was used to identify moderate to severe depression^11^. In the univariate analysis, the dependent variables included mean scores from the STAI-S and BDI-II, and the independent variables included the categorical pain intensity rating, duration of cancer pain, Pain Management Index (PMI) value, and socio-demographic and clinical variables. Candidate variables were identified through exploratory univariate analysis and subsequent multivariate regression analysis. Pain interference and pain relief percentages were not included in the analysis, as there was collinearity between pain intensity and these two variables (Supplementary Table 1: each variable is significant, tolerance is less than 0.1, and the variance inflation factor (VIF) is more than 10).

A one-way ANOVA was used to identify possible predictors among categorical variables. Correlation coefficients were used for continuous, ordinal, and binary variables. A predictor was considered to be a candidate if it exhibited at least a marginal association (*p* ≤ 0.25 and/or r ≥ 0.25). Multiple linear stepwise regression analyses were employed to determine the impact of pain-related variables on anxiety or depression when other identified predictors were controlled (significance was defined by a 95% confidence interval for entry and a 90% confidence interval for removal). Residual diagnostic tests were performed to evaluate the appropriateness of the selected regression models.

## Results

### Patient-Reported Outcome Measures

#### Chinese version of the Brief Pain Inventory (BPI-C)

Cancer pain was measured by the BPI-C, a Chinese version of the validated tool. Coefficient alphas for the pain severity and pain interference items were 0.89 and 0.92, respectively^4^. The BPI included two important domains: pain intensity and pain interference in daily functions. The pain intensity domain consists of four 0 to 10 numeric rating scales (NRS) that ask patients to rate the “worst,” “least,” “average,” and “now” (current) pain in the past 24 hours, where 0 indicates “no pain” and 10 corresponds to the “worst pain imaginable.” Seven items were designed to measure pain interference with general activity, mood, walking ability, normal work, relations with others, sleep, and enjoyment of life.

In this study, the percentage of pain relief and analgesics received were assessed, and the duration of cancer pain for each patient with pain was also recorded. Additionally, the adequacy of pain management was assessed with the PMI in order to evaluate the severity of cancer pain with the category of analgesics prescribed for treatment. Each patient’s PMI value ranged from −3 to +3 and was classified into two categories: values < 0 indicated under or inadequate pain treatment and values ≥ 0 indicated acceptable treatment^12^,^13^.

#### Chinese State-Trait Anxiety Inventory (C-STAI)

Anxiety was measured using the Chinese version of the STAI. The STAI^14^ consists of two subscales: “state anxiety” (STAI-S), or the transitory emotional response to a stressful situation, and “trait anxiety” (STAI-T), or the relatively stable and long-standing disposition to respond to stress with elevated anxiety and a tendency to perceive a wide range of situations as personally threatening. Each subscale comprises 20 items rated from 1 to 4. Scores for each subscale are summed and range from 20 to 80. Coefficient alphas were reported to be 0.90 and 0.81 for the Chinese STAI-S and STAI-T, respectively^15^. Different cutoff values have been used to define anxiety in some studies^16^,^17^,^18^,^19^, but they were not applied in the present study due to the absence of sensitivity and specificity data in a Chinese population.

#### Chinese version of the Beck Depression Inventory (BDI-II)

The BDI-II, the revised version of the BDI-lA, was published in 1996^20^. The score from the 21-item BDI-II scale ranges from 0 to 63. Excellent psychometric properties of the BDI-II have been demonstrated with patients with cancer. Using a cutoff point of 14, the scale showed a sensitivity of 90% and a specificity of 86%^21^. The Chinese version of the BDI-II has been verified to have high internal consistency (alpha = 0.93) with Chinese patients with cancer^22^ and was used to assess depression in this study.

### Sample Characteristics

We observed a significantly poorer ECOG PS for the pain group (*p* = 0.03), and a significantly higher proportion of patients who were not currently receiving anticancer treatment (*p* = 0.01) (Table 1).

### Cancer Pain and the Adequacy of Pain Treatment

In the pain group, 62.5% of patients experienced severe pain (40/64), 15.63%) had moderate pain (10/64), and 21.88% indicated mild pain (14/64).

Pain severity/interference and the percentage of pain relief for the pain group are presented in Table 2. General activity, mood, normal work, and sleep were moderately affected by pain. Only 48.44% (31/64) of patients achieved over 50% pain relief and the mean percentage of pain relief was 46.95% (95%CI: 40.72% to 59.18%).

The average duration of cancer pain was 15.64 weeks (range: 1 to 49 weeks, SD = 11.99; 95%CI: 19.16 to 12.11). Almost half (48.44%) of patients had cancer pain for less than 12 weeks, 34.38% for less than 6 months, and 17.19% for more than 6 months.

Analgesics administered to patients are listed in Table 3. In this study, 90.63% (58/64; 95% CI: 97.76% to 83.49%) of patients with pain were taking analgesics. Oxycodone was the most commonly prescribed opioid. Oral pain medication was given to 84.48% of patients, 81.03% were given analgesics around the clock (ATC), sustained/controlled released analgesics were given to 74.14% of patients, and only 18.97% of patients received drugs on an as-needed basis.

Additionally, the PMI scores calculated were −3 for 2 patients, −2 for 4 patients, −1 for 10 patients, 0 for 35 patients, 1 for 3 patients, and 2 for 10 patients. No patients had a PMI value of 3 which might indicate that no patients had complete pain relief. In summary, 25% (16/64) of patients had a negative PMI, and 75% (48/64) had a PMI score of ≥0.

### Impact of Cancer Pain on State Anxiety and Depression

As shown in Table 4, the pain group had significantly worse state anxiety (STAI-S, *p* = 0.0001) and depressed mood based on the Beck Depression Inventory II (BDI-II, *p* = 0.01) than did the pain-free group. However, there was no observed significant difference in trait anxiety between the two groups. The prevalence of depression, as measured by the BDI-II with a cutoff value of 14, was significantly higher in the pain group (45.31%, 29/64) than in the pain-free group (25.81%, 16/62; OR = 2.38, 95%CI: 1.12 to 5.05, *p* = 0.03).

The impact of pain on these two symptoms was studied further using univariate analysis (Table 5). In patients with pain, the analysis revealed that both state anxiety and depression were not related to patient characteristics (age, gender, ethnicity, marital status, and occupations) or disease factors (cancer type, original stage and current status of disease, and the adequacy of pain management by PMI value). However, state anxiety severity and BDI-II scores were significantly correlated with ECOG PS (*p* = 0.002), duration of pain (*p* = 0.017 and *p* = 0.007, respectively), and the worst level of pain intensity (*p* = 0.000, *p* = 0.013).

Multiple linear stepwise regression analyses were performed to examine the psychological impact of pain, measured by the patients’ reported anxiety state and depression and the results are summarized in Table 6. ECOG PS, anticancer treatment, duration of pain, and the worst level of pain intensity were identified in the final regression model of SA, accounting for 59% of the total variance. The worst level of pain intensity was the most important predictor in the model explaining 41% of the total variance. In the regression model for depression, 30% of the total variance was explained by the average level of pain intensity and duration of pain, as well as by the ECOG PS.

Tolerances for all independent variables ranged from 0.91 to 0.99. Therefore, multicollinearity did not exist in the two models that were used. Standardized residuals were normally distributed, indicating that there was no discernible pattern of correspondence between the residuals and predicted values. These features were consistent with the assumptions of the linear regression model^23^.

## Discussion

### Cancer Pain and Management

In this study, over 60% of patients in the pain group reported severe pain. The levels for the worst and the average pain intensities experienced by the pain group remained moderate. Pain had a moderate effect on four of the seven interference items and only 47% of participants indicated pain relief. However, more than 90% of patients in the pain group were given analgesics. Among them, 80% were taking strong opioids and 81% received analgesia ATC. Additionally, only a quarter of patients had a negative PMI score. The proportion of negative PMI scores in our study is similar or better than that indicated by two recent multicenter surveys^1^,^11^. Overall pain control was also compared with a previous study^4^. Despite the greatly increased PMI values and significantly higher proportion of ATC analgesics, the proportion of severe pain was not significantly lower (70% vs. 62.5%).

A seemingly acceptable proportion of negative PMI scores with relatively low patient-reported percentages of pain relief shown in this study led to the reconsideration of the role of the PMI in evaluating pain management and the reasons for inadequate pain control. Given the subjective nature of pain, we believe patient-reported pain relief is the “gold standard” in evaluating pain management. We also agree that negative PMI values always indicate the undertreatment of pain. Studies have shown high congruence between these two variables, but positive PMI scores do not signify adequate pain control^24^,^25^,^26^. Often, positive PMI values are described as “acceptable” treatment or a preliminary judgment of pain management. In other words, the PMI corresponds to the WHO pain ladder. Therefore, evaluation of pain management requires the combination of multiple evaluation tools together.

From this point of view, most cancer pain in the present study was not effectively controlled. Two factors might have resulted in the uncontrolled cancer pain. First is inadequate opioid dose. While a majority of patients in the pain group were prescribed strong opioids, a rational dose escalation was not obviously achieved. The second factor is the underutilization of adjuvant analgesics. Cancer pain is multifactorial and often involves inflammatory and neuropathic components and, therefore, the appropriate use of adjuvant agents is necessary to mitigate the pain. However, most physicians lack experience in the use of these drugs. In Mainland China, these two factors are widespread^27^ and need to be addressed and improved further.

Several further issues exist that hinder the adequate use of analgesics in China, such as generally low levels of patient compliance for analgesics, a lack of specialized palliative care teams, and a very complicated procedure for opioid prescription. The undertreatment of cancer pain in this study emphasizes the importance of reassessing cancer pain and analgesic regimes, including frequency and dose adjustment. The barriers to adequate pain management also need to be reviewed and gradually overcome.

### Impact of Cancer Pain on State Anxiety and Depression

The present study also investigated the influence of cancer pain on anxiety and depression. The relationship between cancer pain and psychological distress has been well studied. Zaza and Baine^28^ conducted a critical review and concluded there was a strong association between chronic cancer pain and psychosocial distress. However, the relationship between cancer pain and psychological distress was not previously studied in Mainland China. The absence of basic data influenced the authors of this study to conduct a cross-sectional survey since a longitudinal study with interventional measures has become mainstream in current research.

In this study, the STAI and BDI-II were applied to assess anxiety and depression. The advantage of these two measurements in evaluating patients with advanced cancer with multiple symptoms has been identified^29^,^30^. Additionally, we believe that in patients with advanced cancer, pain is always changing; the STAI-S can best describe transient anxiety due to this ever-changing pain.

The present study indicates that, compared to pain-free patients, patients with cancer pain had significantly higher levels of both state anxiety and depression, but not trait anxiety. Furthermore, the prevalence of depression was also significantly higher in patients with pain. Considering the pain status of the participants, the results indicate that uncontrolled pain was a significant risk factor of state anxiety and depression for patients with cancer in Mainland China. Effort has been made to find a controlled study in publication using the STAI and BDI-II simultaneously, without success. Thus, a direct comparison of the levels of state anxiety and depression of patients from the two groups in this study with those from previous publications was not possible. However, an uncontrolled study employing the same pain and psychological measurements as those in our study identified similar levels of anxiety and depression in American patients with cancer pain^31^.

The necessity of a control group was demonstrated in our study by the pain-free patients reporting some degree of anxiety and depression. The levels of state and trait anxiety of the pain-free group were in agreement with previous studies^8^,^32^, and the prevalence of depression of the pain-free group was within the range (10% to 39%) of earlier reports^33^,^34^.

In this study, the impact of cancer pain on state anxiety and depression was further clarified. The worst and average pain intensity levels were identified as significant predictors for state anxiety and depression. Based on the degree of variance explained in each regression model, pain intensity correlated much better with anxiety than with depression. This finding was supported by Chung and Tso’s report^35^. In interpreting the results, transient anxiety is likely to be induced by a patient’s current symptoms, for instance, uncontrolled pain or breakthrough pain. In contrast, depression is a relatively stable symptom that may be caused by a cluster of ongoing or chronic factors.

In this study, both anxiety and depression could be predicted by the duration of cancer pain and the patient’s ECOG PS. These findings in Chinese patients with cancer are consistent with the previously reported negative correlation between the length or duration of cancer pain and psychological distress^36^,^37^. The predictive role of performance status (PS) in anxiety and depression in patients with cancer has been identified^33^,^38^,^39^. PS is often recognized as a functional capacity index that is not only related to patients’ age and disease stage, but also with the concurrence of symptoms. The ECOG PS is a brief and powerful tool that should be implemented to assess psychological distress.

In this study, anticancer therapy was a significant predictor of SA. It is noted that this variable and the ECOG PS differed significantly between the pain and pain-free groups. These results indicate that cancer pain decreased patients’ quality of life and may have prevented them from receiving anticancer therapy. Therefore, multicollinearity between pain-related factors and these two variables should be tested. However, the independent variables in the final regression model were not collinear, based on the collinearity diagnostics. This study indicated that cancer pain is a key factor negatively associated with the ECOG PS, not exclusive to patients with advanced cancer. We also believe the subjective feeling of not receiving anticancer therapy may induce an anxious reaction regarding the disease progression or future life expectancy, possibly explaining the negative influence on patients’ anxiety.

The impact of other previously identified socio-demographic predictors^40^,^41^,^42^, such as age, gender and marital status, were not confirmed in our study. These inconsistencies may arise from differences in patient populations, statistical methodology, and measured symptom domains.

In this study, there were several limitations. Initially, these findings are mainly applicable to Chinese patients with cancer pain experiencing symptoms associated with depression and anxiety. These findings are not representative of patients in other cultures or different regions or locations. Moreover, the use of convenience sampling and the relatively small sample size limit the generalizability of these findings. The potential for sampling bias may exist from the study design as patients were selected based on a specific list of criteria. The participants of the two groups were taken from a limited sample and may not be representative of the entire population. Additionally, other potentially relevant correlates, such as concurrence symptoms, social supports and coping strategies for pain were not analyzed together. The time between intervals of pain in patients might have introduced recall bias in understanding their own pain, depression, and anxiety relative to the cancer pain. In addition, the pain-free group was not evaluated with the ABI-II. Heterogeneity existed between the specific cancer diagnoses received by patients and this may have played a role in the pain, anxiety, and depression responses.

Furthermore, the neoplasm stage was not taken into account. Therefore, the variance of SA and depression could only be partially explained by the identified variables. There are some limitations to the study design, particularly in that relying on patient-reported anxiety and depression scales can present a less objective method to quantify and rationalize data, resulting in less statistical significance. Moreover, the methods to screen for anxiety and depression in patients were not diagnostic but symptomatic evaluations. In this respect, some of the patients might not have had a clear diagnosis of anxiety or depression. However, the results of the study did present the current situation of cancer pain control and extend previous findings regarding the impact of cancer pain on anxiety and depression in Chinese patients.

## Conclusion

In conclusion, despite the relatively low proportion of negative PMI scores, cancer pain was not effectively treated. Compared with the pain-free group, the pain group demonstrated significantly lower ECOG PS, fewer patients receiving anticancer treatment, and higher means STAI-S and BDI-II scores. Predictors of SA and depression included the worst and average levels of pain intensity, duration of cancer pain, ECOG PS, and whether or not anticancer therapy was received. With respect to the proportion of total variance, the worst pain intensity level played the most important role in predicting SA. The frequent assessment of cancer pain and the adjustment of analgesic regimes are critical. Based on these findings, we believe psychological screening should be routinely performed with patients experiencing cancer pain, with poor ECOG PS, and who are not receiving anti-cancer treatment.

## Additional Information

**How to cite this article**: Li, X.-M. *et al*. Psychological distress and cancer pain: Results from a controlled cross-sectional survey in China. *Sci. Rep.* **7**, 39397; doi: 10.1038/srep39397 (2017).

**Publisher's note:** Springer Nature remains neutral with regard to jurisdictional claims in published maps and institutional affiliations.

## Supplementary Material

Supplementary Information

## Figures and Tables

**Table 1:** Socio-demographic and clinical characteristics of the two groups.

Characteristic	Patients with pain n = 64	Patients without pain n = 62	*P value*
**Mean age in years (SD)**	64.19 (11.21)	63.36 (10.17)	0.66
**Gender**			0.98
Male (%)	38 (59.38)	36 (58.06)	
Female (%)	26 (40.63)	26 (41.94)	
**Ethnicity**			0.75
Han (%)	60 (93.75)	58 (93.55)	
Other (%)	4 (6.25)	4 (6.45)	
**Marital status**			0.96
Married (%)	46 (71.88)	47 (75.81)	
Single (%)	4 (6.25)	3 (4.84)	
Divorced (%)	8 (12.50)	7 (11.29)	
Widowed (%)	6 (9.38)	5 (8.06)	
**Highest education level**			0.86
≤Junior high school (%)	21 (32.81)	18 (29.03)	
Senior high school (%)	25 (39.06)	27 (43.55)	
College and graduate school (%)	18 (28.13)	17 (27.42)	
**Occupation**			0.34
Agricultural	9 (14.06)	3 (4.84)	
Factory	11 (17.19)	14 (22.58)	
Professional/sales	21 (32.81)	21 (33.87)	
Retired/other	23 (35.94)	24 (38.71)	
**Cancer type**			0.90
Lung	21 (32.81)	19 (30.65)	
Colon and Rectal (Combined)	14 (21.88)	12 (19.35)	
Breast	13 (20.31)	16 (25.80)	
Others	16 (25.00)	15 (24.19)	
**Stage of disease at diagnosis**			0.29
Stage I-II (%)	12 (18.75)	17 (27.42)	
Stage II-III (%)	52 (81.25)	45 (72.58)	
**Current metastatic sites**			0.29
No site Single site	10 (15.63)	16 (25.81)	
	11 (17.19)	12 (19.35)	
Multiple sites	43 (67.19)	34 (54.84)	
**Current ECOG PS**#			
0–1	15 (23.44)	27 (43.55)	0.03
≥2	49 (76.56)	35 (56.45)	0.03
Mean (SD)	2.23 (1.07)	1.86 (0.83)	
**Currently receiving anticancer treatment**			0.01
Yes (%)	36 (56.25)	49 (79.03)	
No (%)	28 (43.75)	13 (20.97)	
**Current status of disease**			0.71
CR	3 (4.69)	4 (6.45)	
PR	8 (12.50)	9 (14.52)	
SD	14 (21.88)	19 (30.65)	
PD	36 (56.25)	28 (45.16)	
Unknown	3 (4.69)	2 (3.23)	

^#^ECOG PS: Eastern Cooperative Oncology Group performance status, a level of 0 indicates full activity without any restriction. Higher levels indicate greater impairment in function.

**Table 2:** Scores of pain intensity/interference and percentage of pain relief (n = 64).

Items	Mean (SD)/Number of Patients (%)	95%CI
**Pain intensity in the past 24 hours**		
The worst	5.88 (2.32)	6.17 to 5.59
The least	3.25 (2.13)	3.5 to 2.98
The average	4.45 (1.92)	4.69 to 4.21
Right now	2.19 (1.73)	2.41 to 1.97
**Pain interfered with daily function**		
General activity	5.23 (2.05)	5.48 to 4.97
Mood	4.17 (2.12)	4.43 to 3.90
Walking ability	3.45 (2.01)	3.70 to 3.19
Normal work	4.34 (1.92)	4.56 to 4.08
Relations with other people	3.41 (1.23)	3.56 to 3.25
Sleep	4.75 (1.85)	4.98 to 4.51
Enjoy of life	3.45 (1.26)	3.61 to 3.29
**Pain relief in the past 24 hours**		
30%	12 (18.75)	13.34% to 24.16%
30–50%	21 (32.81)	30.08% to 36.31%
51–80%	22 (34.38)	51.62% to 60.89%
80%	9 (14.06)	80.14% to 81.57%
Average	46.95%	34.72% to 59.18%

**Table 3:** Analgesics currently taking by pain patients (n = 58).

Analgesic Medications	Number of Patients (%)	95%CI
**Strong Opioid**	45 (77.59)	67.37% to 87.81%
Oxycodone	35 (60.34)	48.35% to 72.33%
Fentanyl	6 (10.34)	2.88% to 17.8%
Morphine	4 (6.90)	0.69% to 13.11%
**Weak Opioid**	5 (8.62)	1.74% to 15.5%
Tramadol	3 (5.17)	0.00% to 10.59%
Codeine	2 (3.45)	0.00% to 7.92%
**NSAIDS and Acetaminophen**	8 (13.79%)	5.34% to 22.24%

**Table 4:** Comparison of anxiety and depression of the two groups.

Variables	Pain group (n = 64)	Pain-free group (n = 62)	t value	P value
Mean STAI-S (SD)	46.38 (8.04)	40.73 (7.13)	4.17	0.0001
Mean STAI-T (SD)	44.64 (7.76)	42.87 (7.67)	1.29	0.20
BDI-II (SD)	19.17 (9.36)	15.35 (7.08)	2.59	0.01

**Table 5:** Variables correlated with state anxiety and depression of the pain patients (n = 64).

Variables	r value	P value
**State Anxiety correlated**		
The highest education level	0.21	0.098
ECOG PS	0.39	0.002
Currently receiving anticancer treatment	0.22	0.077
Length of cancer pain, weeks.	0.30	0.017
The worst PI in the past 24 hrs	0.64	0.000
The average in the past 24 hrs	0.52	0.000
**Depression correlated**		
The highest education level	0.16	0.217
ECOG PS	0.37	0.002
Number of metastatic sites	0.19	0.136
Length of cancer pain, weeks.	0.33	0.007
The worst PI in the past 24 hrs	0.31	0.013
The average in the past 24 hrs	0.37	0.003

**Table 6:** Multiple regression models for predictors of state anxiety and depression (n = 64).

Predicting variables	R^2^	R^2^ (change)	Standardized Coefficients
**State Anxiety**			
The worst PI in the past 24 hrs	0.412	0.412	0.567^c^
Currently receiving anticancer treatment	0.472	0.060	0.268^a^
Length of cancer pain, weeks.	0.537	0.064	0.249^a^
ECOG PS	0.587	0.051	0.233^a^
**BDI- II**			
ECOG PS	0.140	0.140	0.294^b^
Length of cancer pain, weeks.	0.238	0.099	0.278^b^
The average PI in the past 24 hrs	0.295	0.056	0.248^b^

^a^p < 0.01; ^b^p < 0.05; ^c^p < 0.001.
