# Supplementary material for: Psychological distress and cancer pain: Results from a controlled cross-sectional survey in China
Source: Sci Rep. 2017 Jan 11;7:39397. doi: 10.1038/srep39397 (PMC5225451; doi:10.1038/srep39397)
Supplement: Supplementary Information [file srep39397-s1.pdf]

Psychological distress and cancer pain: Results from a controlled cross-sectional survey in China

Running head: Psychological distress and cancer pain in Chinese-cancer-patients

Xiao-mei Li[1,*], Wen-hua Xiao[2], Ping Yang[3], Hui-xia Zhao[2]

[1]Department of Medical Oncology, Chinese PLA General Hospital, Beijing, No.28, Fuxing Road, Haidian district, Beijing 100853, China;

[2]Department of Medical Oncology, the First Affiliated Hospital of Chinese PLA General Hospital, Beijing 100048, China;

[3]Department of Medical Oncology, PLA Navy General Hospital, Beijing 100048, China.

[*]**Corresponding author:**

Dr. Xiaomei Li

Department of Medical Oncology, Chinese PLA General Hospital

No.28, Fuxing Road, Haidian district, Beijing 100853, China

Tel: +86-10- 68182255, Fax: +86-10- 68182255

Email: lixiaomei201306@126.com

Supplementary Table 1. Collinearity test among pain intensity, pain interferences and pain relief percentages

Variables	Tolerance	Variance inflation factor (VIF)	P value
Pain intensity	0.083	12.121	<0.05
Pain interferences	0.069	14.546	<0.05
Pain relief percentages	0.047	21.325	<0.05
